# Strengthening Tobacco 21 implementation and enforcement to reduce tobacco-related health disparities: A stakeholder engagement project

**DOI:** 10.18332/tpc/163299

**Published:** 2023-06-16

**Authors:** Summer Woolsey, Athena K. Ramos, Kaeli Samson, Delwyn Catley, Keyonna M. King, Rob Crane, Hongying Daisy Dai

**Affiliations:** College of Public Health, University of Nebraska Medical Center, Omaha, United States; The Center for Children's Healthy Lifestyles and Nutrition, Children’s Mercy Hospital, Kansas City, Missouri; Preventing Tobacco Addiction Foundation, Dublin, Ohio

**Keywords:** Tobacco 21, implementation, enforcement, compliance, equity, focus group

## Abstract

**INTRODUCTION:**

As a part of a priority-setting stakeholder engagement project to strengthen the impact of the federal Tobacco 21 (T21) law, we conducted a qualitative study to solicit input from a nationwide sample of tobacco control stakeholders regarding the implementation, enforcement, and equity implications of the T21 law.

**METHODS:**

Following the T21 policy evaluation guidance developed by the Centers for Disease Control (CDC), we identified T21 experts in four domains: policy, evaluation, subject matter, and implementation from a national search of stakeholders (invitations, n=1279) to account for geographical variation. This study presents results from five focus groups conducted in December 2021 among stakeholders (n=31) with experience in T21 policy, evaluation, subject matter, and implementation.

**RESULTS:**

Participating T21 stakeholders reported on eight themes from four main topic areas: 1) Implementation, 2) Enforcement, 3) Equity outcomes, and 4) Recommended changes from stakeholders. Stakeholders shared insights on both passive and active implementation methods used in their communities, and highlighted major barriers such as the absence of a standardized tobacco retail licensing mandate and insufficient resources. Regarding T21 enforcement, stakeholders believed that current deterrents for retail violations might not be effective. They noted that vape and tobacco shops and online sales of tobacco products are emerging major challenges in T21 enforcement. Stakeholders also discussed possible health inequities that may be exacerbated by heterogenous implementation of the T21 law.

**CONCLUSIONS:**

To strengthen T21 and mitigate potential exacerbation of existing health inequities, greater alignment of federal, state, and local efforts to reduce heterogeneity of implementation and enforcement of the T21 law is recommended.

## INTRODUCTION

The federal Tobacco 21 (T21) law, which raised the minimum legal age of sale (MLSA) of any tobacco product from 18 to 21 years was signed in December 2019^1^. As a result of efforts from an influential grassroots ‘Tobacco 21 Movement’, localized T21 laws were adopted prior to the national law in 19 US states, including the District of Columbia, two US territories and over 540 localities^2,3^. The National Academy of Sciences estimates that the federal T21 law will prevent 223000 deaths by delaying youth tobacco use initiation^4^.

Most existing literature on T21 implementation, enforcement and outcomes has centered around the effectiveness of T21 policy with mixed results^5-13^. Researchers have raised concern that the potential public health benefits of the law might be weakened by deficiencies in enforcement measures. Further research has found that MLSA restrictions are ‘not likely to be effective without significant age-verification requirements and increases in the number of and frequency of compliance checks that the FDA conducts’^14^.

There is a growing body of literature on best practices, assessment tools and policy logic models aimed at improving T21 outcomes^13,15,16^. To begin assessing adherence to model policies across the nation, Dobbs et al.^13^ created a T21 policy assessment tool to evaluate the language used in state T21 implementation and enforcement laws. Their study found that across 16 US states, laws ‘varied widely’ in terms of ‘key policy components’ such as inspection policies, penalties for retail violations, and retail licenses. As the federal law does not mandate specific enforcement measures, such as the number of times a retailer is checked, more research is needed to understand how to best strengthen the T21 law across the nation to reach the maximum effect.

Along with the overall impact on underage tobacco use, the potential impact of T21 on health equity has also been the subject of an emerging body of research. While the ‘piecemeal passage of T21 laws at the local and state levels was cause for concern from a health equity perspective’, researchers note that the national T21 law has the potential to reduce tobacco use disparities throughout the nation^17^. Although the federal T21 law does not include provisions about Purchase, Use, and Possession (PUP) laws, which penalize minors for possession of a tobacco product, nor how states enforce T21 under civil or criminal law, both areas are causes for concern in terms of exacerbating inequities among communities that have been historically over-policed and have disproportionate levels of tobacco use^16^.

While great strides are being made in the attempt to strengthen T21, more research is needed to understand the barriers experienced by stakeholders to implement and enforce T21 in their localities. Voices of stakeholders are necessary for understanding the unique challenges faced in each locality and identifying potential resources to strengthen T21. The purpose of this study is to add to the existing literature on T21 implementation, enforcement and outcomes through exploring the viewpoints of a nationwide group of T21 stakeholders.

## METHODS

### Stakeholder engagement project

We followed the policy evaluation guidelines developed by the Centers for Disease Control and Prevention (CDC)^18^. This stakeholder engagement project leveraged multiple methods, including a quantitative survey (n=246, Stage 1) and qualitative analysis of focus groups with stakeholders (n=31, Stage 2) and the general public (n=80, Stage 3) as three separate projects. This article presents Stage 2 results from five focus groups conducted in December 2021 with stakeholders (n=31) who had experience in T21 implementation and enforcement and who had completed the survey regarding T21 practices in their communities.

### Sample recruitment

To ensure that the sample included T21 stakeholders with knowledge of and experience with the federal T21 law, we used a purposive sampling strategy. The research team conducted an extensive online search for tobacco coalitions, T21-related organizations, prominent national tobacco control conferences, and tobacco control policy research symposiums to identify stakeholders. In October 2021, our team sent 1279 initial invitation emails asking participants to complete a survey on their opinions of various tobacco control policies. Individuals identified as stakeholders in one or more categories were invited to participate in the study, including policy experts, evaluation experts, subject matter experts, and implementers (Supplementary file Table 1). Of the 1279 identified stakeholders initially contacted, 246 (19.2%) provided responses for the quantitative survey in October 2021. At the end of each survey, participants were invited to participate in a follow-up focus group discussion about their attitudes on T21 implementation, enforcement and outcomes via video conferencing software. Based on the results of Stage 1, only a small percentage of experts reported that they worked closely with T21 policies. By leveraging this sequential study design, we identified a small number of T21 experts from a large national sample and avoided contaminating our findings with experts unfamiliar with T21 policies. Of the 246 stakeholders who completed the survey, 34 (13.8%) indicated their interest and scheduled a time to participate.

### Focus group procedure

This qualitative study used focus groups to collect stakeholder attitudes and knowledge of T21 implementation, enforcement, and outcomes. Five focus groups were held in December 2021, each lasting approximately one hour. Of the initial 34 stakeholders who signed up to participate in a focus group, 31 attended, with a median focus group size of six participants and a range of 4–8 participants in each focus group. Participants represented a variety of T21 stakeholder categories and geographical locations within the US. All focus groups were conducted and recorded via virtual conferencing software and facilitated by two research team members with relevant training (DC and SW), using a semi-structured interview protocol. The research team developed the interview protocol to address the following key domains: T21 implementation, enforcement, retailer compliance, and community impacts. Two other research team members (HD and KS) were present for each focus group to take detailed notes and ask additional follow-up questions. (Supplementary file Tables 2 and 3) for sample characteristics, detailed focus group procedure, and interview constructs.

### Analysis

All focus groups were recorded and transcribed by a professional third-party transcription service, ‘Transcribe Me’^19^. To ensure the quality of the transcriptions, a research team member checked each transcript for accuracy while watching recordings of each focus group. Minimal changes to correct spelling or instances of cross-speak were made. Then, two members of the research team read through each transcript to develop an initial codebook. Afterward, they independently coded all transcripts and returned to review each for agreement, resolve inconsistencies, and update the codebook. Another coding pass was completed using the updated codebook to conduct a thematic analysis of the data. These themes were presented to other members of the research team to discuss and review evidence associated with each theme. We employed the Consolidated Criteria for Reporting Qualitative Research (COREQ) framework (Supplementary file Table 4) to present study findings. Data for the initial survey where participants expressed interest in participating in a focus group were collected and managed using research electronic data capture (REDCap) tools^20,21^ hosted at University of Nebraska Medical Center.

## RESULTS

A total of 31 participants from across the nation attended five online focus groups in December 2021. Focus group participants were from 16 different US states and were assigned to a focus group based on scheduling convenience (Supplementary file Table 2). Participants had the option to self-identify in more than one T21 stakeholder category. As a result, 17 participants identified as subject-matter experts (54.8%), 12 as policy experts (38.7%), five as evaluation experts (16.1%), four as implementation staff (12.9%), and four as ‘other’ (12.9%).

Eight themes resulted from the analysis of four T21 topical areas: 1) Implementation, 2) Enforcement, 3) Equitable outcomes, and 4) Recommended changes from stakeholders.

### Tobacco 21 Implementation

Table 1 shows two themes associated with T21 implementation.

**Table 1:** Themes for Tobacco 21 implementation, as discussed by focus groups of stakeholders

*Theme*	*Code*	*Example quotes from each focus group (FG)*
**Passive and active education strategies**	**Passive strategies**	**Signage** *‘I did notice most of the signage was company-created. So if they were a small mom-and-pop, they printed it out on their computer. If they were QuikTrip or Casey’s, something came down from their corporate level.’ (FG 4)*
		*‘I’ve noticed quite a few more signs, signs outside of these retailers, a gas kiosk. Places are now sporting signs indicating age requirements, and that there will be asking for ID and that sort of thing.’ (FG 3)*
		**Toolkits and other educational material** *‘And it’s called a Tobacco 21 toolkit … the kit is a way to remind the store clerk. Well, the store owner, to remind his clerks to check for IDs. It’s got ID pins. It’s got frequently asked questions about tobacco, and about Tobacco 21. If there are any misconceptions about the law. It’s got some signage. Which is actually required by Kentucky law, but I’m not seeing much of it up.’ (FG2)*
		*‘[A community-based youth group] did develop these basically printed business cards, and it was when you go into a gas station or a retailer, pretty much a gas station or a grocery store, that you could hand it to the retailer and say, “I care about Tobacco 21. I care about kids in our community. And just thank you for upholding this.’ (FG 5)*
	**Active strategies**	**Personal letters to retailers** *‘… we sent out personal letters to all of the license holders, letting them know of the law change and what to expect,and offered resources if they needed anything for their store signage or anything like that. Specifically, we also have on hand that we have handed out that this is our watch materials that we could obtain for free from that program.’*
		**Retailer training** *‘So our department of revenue and department of public health and environment have collaborated for a fair amount of time, years actually, around merchant education. They send signs, they send age calculators, they have a how-to website. The department of revenue’s tobacco website has all the laws.’ (FG 3)*
		*‘It was first education by the legal resource center to our health departments. And then our health departments, who are the ones that have direct contact with our retailers, were the ones who were doing a lot of retailer education, both in person and electronically.’ (FG 4)*
**Significant barriers to Tobacco 21 implementation**	**Funding and resource limitations**	*‘I would love to see is something that’s coming down from either FDA, CDC, or state health department or state tobacco control. Coming down to the communities, saying, “Hey, here’s a nice little packet that you can take out. Here’s how you have those conversations”. And less of, “Yeah, it’s your community, you figure out what they want to know”. Because there’s not enough resources down at the local level.’ (FG 4)*
		*‘I know a lot of communities wouldn’t be able to afford that, even providing one sign at $10 to 200 people … or 200 stores.’ (FG 4)*
	**Lack of state-wide tobacco retail licenses**	*‘We deliver materials to every tobacco retailer that we're aware of because we don't have a state list of tobacco retailers either. But all the retailers that we can find and be made aware of from other different state lists from … it's kind of a mess how they put it together.’ (FG 1)*
		*‘I would say that when it came time for implementation in our community, we realized how much it hurt us that we did not have a statewide Tobacco retailer license, and how much that licensing process helps with implementation.’ (FG 5)*
	**Challenges in retailer training**	*‘So maybe there is a need to produce an education video or some kind of education that retailers can use with all the new clerks that they're constantly hiring that may work there two weeks and then go to another job, and they've got to replace that employee.’ (FG 1)*
		*‘My takeaway on that is merchant education can be effective if the store owners make it part of their culture and stress that importance to their employees. That is the biggest challenge in my work is trying to create that spark on the owner to get them to make it part of their store policy. It unfortunately doesn't happen as much as I would like it to.’ (FG 2)*
	**Complexities from competing state and local laws**	*‘And I felt like one of the things we noticed was there was a lot of that confusion because Nebraska felt very strongly that we were just going to … state law superseded federal. So they said, “We had just moved it to 19. They can’t tell us what to do, so we’re going to stay at 19”.’ (FG 4)*
		*‘But at the same time, we’re instructed to … while we’re handing out stickers that say 21 and under like WE Card materials, FDA our watch materials, like the calendars and everything, we have to hand out the state law sign that says 18 and under. So it’s very, very confusing.’ (FG 3)*
	**Current public awareness and education of Tobacco 21**	*‘And I’m just like, “Oh, it’s still in the works”, because I haven’t heard anything just from a general public perspective the little bit of social media or TV that I do watch. I don’t see any commercials, or advertisements, or anything that’s like, “Hey, guys. This is the new thing. You have to be 21 to purchase a tobacco product”. And so I don’t feel like the message is out there’. (FG 3)*
		*‘I know that there was some education to retailers on the new policies when Tobacco 21 passed. But to the general public, there was not a very robust communications plan.’ (FG 5)*

*Theme 1: Passive and active education strategies*

Participants reported a variety of strategies used to raise awareness and educate about the passage of the federal T21 law. Although the majority of stakeholders described the importance of both passive and active strategies to reach this goal, most stakeholders reported the use of passive strategies that required minimal to no contact with retailers or the public, such as posting and distributing signage to tobacco retailers, disseminating retailer toolkits, and sharing written educational materials, such as brochures.

Active strategies that required face-to-face or virtual contact with retailers or the public included retailer education efforts and attempts to directly reach out to tobacco retailers. Retailer education efforts varied regarding how education programs were delivered, when they were offered, and by whom. Stakeholders named local health departments, local governmental offices, and local law enforcement agencies as the organizations responsible for retailer education in their respective communities.

*Theme 2: Significant barriers to Tobacco 21 implementation*

Funding and resource limitations

Most stakeholders cited a lack of funding and resources as a common barrier to T21 implementation in their communities for both the passive and active strategies mentioned above (Focus group 4).

Lack of state tobacco retail licenses

Many stakeholders also highlighted the absence of a statewide licensing system as a common barrier to T21 implementation, as stakeholders were unable to access a comprehensive list of tobacco retailers in their area. Stakeholders had to compile and rely on lists of retailers that were often incomplete or possibly outdated due to fluctuations in the market. As one stakeholder described:

*‘It’s kind of a mess how they put it [different state lists of tobacco retailers] together.’* (Focus group 1)

Accessibility and format of retailer trainings

Challenges associated with tobacco retailer education efforts included accessibility and format of retailer training. A few stakeholders described a need for virtual or digital options in addition to in-person training. Business owners were challenged with high employee turnover rates and ensuring adequate training for their staff. Virtual or digital options could be helpful. One participant noted:

*‘So maybe there is a need to produce an education video or some kind of education that retailers can use with all the new clerks that they’re constantly hiring …’* (Focus group 4)

When online training was available, several stakeholders also noted that retailers were facing issues with engagement and accountability, stating that it was difficult to tell if the employees were engaging with the material or simply clicking through each section. A final challenge discussed in retailer training was a lack of business owners making training part of their culture and stressing its importance to their employees (Focus group 3).

Competing state and local laws

Another significant barrier described by stakeholders to T21 implementation was the complexities caused by competing state and local laws regarding MLSA. Several stakeholders lived in a state in which the state tobacco MLSA was not 21 years but typically 18 years, which created confusion for the public, tobacco retailers, and stakeholders. Participants referenced instances in which communities believed that state and local laws overruled the federal T21 law, as described by one stakeholder from Nebraska (Focus group 4).

Lack of awareness and education among the general public

A final implementation barrier described by a majority of stakeholders was a lack of awareness and education of the T21 law among the general public. Stakeholders reported an absence of public-facing communications about T21 in their communities. In discussing the federal T21 law, one stakeholder noted:

*‘I don’t see any commercials, or advertisements, or anything that’s like, “Hey, guys. This is the new thing. You have to be 21 to purchase a tobacco product”.’* (Focus group 3)

### Tobacco 21 Enforcement

Table 2 summarizes three themes associated with T21 enforcement with example quotes from stakeholders.

**Table 2:** Themes for Tobacco 21 enforcement, as discussed by focus groups of stakeholders

*Theme*	*Code*	*Example quotes from each focus group (FG)*
**Variation in enforcement measure protocols**	**Age of cooperating individuals for compliance checks vary by locality**	*‘We complete compliance checks ... And our officer used a 15-year-old. They’re still using lower age individuals in our area.’ (FG 1)*
		*‘So we just started using the 18 to 20-year-olds two years ago when Tobacco 21 went into effect.’ (FG 2)*
		*‘So some people are still using under 18 … or I mean, some jurisdictions are still using under 18, but some did choose to go a little bit higher once Tobacco 21 became effective.’ (FG 4)*
	**Frequency and distribution of compliance checks vary**	*‘I think Colorado might do it twice a year, there’s a handful of states that do twice a year, once a year minimum with a 24 to 36 month look-back period.’ (FG 2)*
		*‘...we just get a small sample-size. And I serve one of the most populated areas, and I can tell you it’s like 168 businesses each year, and it’s for just one check.’ (FG 3)*
		*‘They do [compliance checks] on a monthly basis, I believe … where all of the retailers within city limits are required by city ordinance to participate in their retailer training which focuses on checking IDs.’ (FG 4)*
	**Compliance check complications due to the COVID-19 pandemic**	*‘And in the past, it was done annually. But it's kind of sketchy out here. It depends on budget cuts. And I also COVID had an impact on it.’ (FG 1)*
		*‘I know that our students have an opportunity about every three months from our school to do a ride along, and I know that they try to get out every month. But I can tell you that with COVID, I know that didn’t happen for 18 or 20 months.’ (FG 5)*
		*‘But part of it was also that there had been no inspections on any of the retailers because of COVID, so there was no enforcement efforts. Retailers, for whatever reason, they got lax. And so when we started them up, boom.’ (FG 2)*
	**Penalties for retail violations vary**	*‘We’re working in Alaska right now, and it’s interesting even though they’re not 21, I mentioned earlier that the first penalty is a 20-day suspension of the license. And when that happened, they saw a significant change because retailers don’t want to lose their right to sell tobacco products.’ (FG 2)*
		*‘If a retailer does sell, they often will be recommended to do a education program funded through the local health department before they get any sort of major fines or anything like that.’ (FG 4)*
	**A variety of entities complete retail compliance checks**	*‘In the state of Missouri, we do … each prevention resource center does the Synar Compliance Survey to a [random] sample of businesses within our region. But we only have in the state of Missouri I think six agents to cover the entire state. We don’t have a lot of funding for it. So more time … more times than not, they don’t really get done. So it’s up to local law enforcement to do those compliance checks.’ (FG 3)*
		*‘I can talk a little bit about Arizona...we have a joint [inspection] program with the FDA.’ (FG 2)*
**Emerging areas of enforcement facing major challenges**	**Difference in tobacco versus vape shops**	*‘When I go and visit tobacco shops and vape shops in my local area, they’re very different in terms of their attitudes toward minimum age requirements. Certainly vape shops are like … in trying to stand out and distinguish vapes from cigarettes, they’re like, “Oh, no. We’re a completely above-board shop. We’re not the tobacco industry and so we absolutely adhere to minimum purchase laws.’ (FG 1)*
		*‘But our Synar visits and our tobacco merchant education does not… if you took it to a vape shop that only sells electronic cigarettes, we do not visit them as a tobacco merchant retailer, because in Missouri, electronic cigarettes are not regulated like other tobacco products.’ (FG 1 )*
	**Online sale of tobacco products**	*‘The online space begins to transcend municipal boundaries and begins to undermine and skirt any sort of regulatory prohibitions on sales, and I think that's a regulatory challenge because for the retailer that is in a jurisdiction that has some sort of age or flavor ban, to make sure that their online sales are happening, but also how do you control retailers or sale of prohibited products from an outside vendor?’ (FG 1)*
		*‘Currently, in social media, they don't have age gatekeeping procedures. So the age gatekeeping procedures should be required and added to the promotion posts for those tobacco companies.’ (FG 3)*
**Current FDA and SYNAR compliance efforts are insufficient**	**Siloed efforts in communities**	*‘We have some coalitions, again, that have tried to dedicate grant funding to doing their own because there's such a problem with the lack of compliance checks that happen. But, again, in the last couple of years with FDA conducting them, there have been more. But that is really separated from the rest of the prevention and enforcement world with the coalition world.’ (FG 1)*
		*‘And if it's a Synar inspection, that's state, so it's a state law. If it's an FDA, then it's a federal, and then you have a whole different system, a different age thing to deal with.’ (FG 2)*
	**FDA and SYNAR compliance check frequency, pattern, and penalties are inadequate**	*‘...we don't have anything that happens to anybody for Synar. For the FDA compliance checks, that's different. But for Synar, it's just like fact-finding I feel like is what it is.’ (FG 1)*
		*‘Yeah, the FDA checks, in my opinion, nobody gets in trouble by the FDA when it comes to tobacco sales. I mean, it's really weak. It's really on the states to actually enforce it and protect their communities.’ (FG 2)*

*Theme 3: Large variation in enforcement measures and protocols*

Discussion around current enforcement measures focused on retail compliance check protocols and penalties when a retailer was found to be in violation of the T21 law. As stakeholders represented a diverse array of communities, responses varied regarding compliance check protocols. The ages of covert buyers (‘decoys’) to conduct retailer compliance checks varied greatly by community. Some stakeholders reported cooperating individuals as young as 15 years old being used to conduct compliance checks (Focus group 1), while others reported having compliance check protocols that utilized individuals aged 18–20 years (Focus group 2).

The frequency and distribution of retail compliance checks differed by community as well. Stakeholders reported annual, biannual, and sometimes even lower frequency compliance checks in their communities. Recurring visits for retailers found in non-compliance also depended on locality. One stakeholder from Colorado outlined their retail compliance policy to be twice a year, but mentioned:

*‘There’s a handful of states that do twice a year, once a year minimum with a 24 to 36 month look-back period.’* (Focus group 2)

At the same time, stakeholders from other localities reported not having a ‘look-back’ policy, a policy in which an establishment with a prior violation is monitored for future violations and issued more severe penalties for repeated violations. Policies regarding frequency and distribution of checks included planned routes that reduced mileage for enforcement personnel, checking all retailers within a jurisdiction, or checking a sample of all retailers within an enforcement agency’s jurisdiction.

Agencies tasked with conducting retail compliance checks depended on the stakeholder’s locality as well. Agencies mentioned in our focus groups included local law enforcement agencies, local health departments, local tobacco control coalitions, FDA contracted partners, and Synar reporting agencies through the Substance Abuse and Mental Health Services Administration (SAMSHA). Stakeholders noted that while each state had FDA contractors and Synar reporters, state and local agencies varied. They noted that penalties for non-compliance or retail violations also depended upon locality. Current penalties reported included monetary fines given to business owners and clerks, loss or temporary suspension of tobacco retail license, or diversion programs in lieu of monetary fines.

*Theme 4: Emerging areas of enforcement facing major challenges*

Stakeholders described two emerging issues as major challenges in T21 enforcement: 1) vape and tobacco shops, and 2) online sale of tobacco products. Vape and tobacco shops are a concern for T21 stakeholders for a number of reasons, including differences in regulation of these shops. As one stakeholder explained:

*‘We do not visit them as a tobacco merchant retailer, because in Missouri, electronic cigarettes are not regulated like other tobacco products.’* (Focus group 1)

Online tobacco sales, including the use of social media, was another emerging concern. Stakeholders commented on the relative ease and access to purchase tobacco among youth and reported limited gate-keeping procedures in the online space, including both online retailers and on social media, to verify the purchaser’s age (Focus group 1). Another concern associated with the online sale of tobacco was the complexity introduced by the online space in terms of who is responsible for enforcing MLSA and how such measures should be enforced.

*Theme 5: Current FDA and SYNAR compliance efforts are insufficient*

When discussing federal efforts to enforce T21, most stakeholders believed that current efforts through the FDA and the SAMHSA Synar program were insufficient, as their efforts were largely siloed in communities and the frequency, pattern, and penalties associated with their compliance checks were inadequate. Many stakeholders reported a gap between the FDA’s enforcement efforts with other agencies involved in T21 enforcement. One stakeholder explained that the FDA compliance inspections did not match the current needs of their community (Focus group 1). Stakeholders believed that penalties were rarely issued by the FDA, and if issued, such efforts were not sufficient to deter future violations. Because of this, the onus of enforcement was often placed upon state organizations, as indicated by one stakeholder:

*‘In my opinion, nobody gets in trouble by the FDA when it comes to tobacco sales. I mean, it’s really weak. It’s really on the states to actually enforce it and protect their communities.’* (Focus group 2)

### Health equity in Tobacco 21 outcomes

Table 3 shows one major theme centered around health equity emerging from discussions around the outcomes of the T21 law.

**Table 3:** Themes for Tobacco 21 equitable outcomes, as discussed by focus groups of stakeholders

*Theme*	*Code*	*Example quotes from each focus group (FG)*
**Stakeholders are concerned about potential implications of Tobacco 21 on health equity**	**Unintended consequence of exacerbating police profiling of marginalized communities**	*‘I will say the thing that I’m most concerned about with enforcement is it seems like some policymakers when they were writing up this legislation thought it would make the law more impactful to include purchase, use, and possession provisions in tobacco 21 laws. And that essentially puts the onus on at least partially on the user, under 18 individuals that are using tobacco products. If there’s any type of fine or punishment or police presence in terms of enforcing that, I think that could have considerable health equity implications considering the police state in America where police disproportionately are likely to pull over Black and Brown individuals or individuals of lower socioeconomic status.’ (FG 3)*
		*In response to if Tobacco 21 can help reduce health inequities: ‘I’d say yes if the law is designed to be a civil issue and not a criminal issue. And again, I know I keep harping on it. I think if the penalty is on the retailer and not on the purchaser, it can help, but if it’s designed to penalize kids who make attempts or have possession, then it’s going to cause issues between the police and kids in marginalized communities that will have another reason to be targeted.’ (FG 2)*
		*‘I mean, in theory, right, we’re saying that if we prevent minority populations from having access to these products or using these products, that theoretically, over the years, their health outcomes will improve because we have less people using these products. What it will look like as we actually roll it out, I think it will be dependent on each community and whether or not they want to use it for profiling.’ (FG 4)*
		*‘My concerns are with the purchase use and possession laws, and that those could be used … particularly in this time during a Black Lives Matters movement, that it could be used to stereotype. It could be used to profile youth.’ (FG 4)*

*Theme 6: Stakeholders are concerned about the potential implications of T21 on equity*

While some stakeholders believed that T21 had the potential to reduce health disparities among groups disproportionately affected by tobacco use by reducing youth tobacco initiation, other stakeholders expressed concern that T21 may have an unintended consequence of exacerbating discriminatory profiling of historically over-policed and marginalized groups. While the federal T21 law does not have provisions for PUP laws, which penalize underage buyers of tobacco products, stakeholders were concerned that localities that have state and local PUP laws would see an exacerbation of enforcement-based profiling in the wake of the T21 law. In addition to this concern, several stakeholders stated that the way penalties are enforced could have considerable impacts on equity, stating that T21 penalties should be enforced as a civil issue, rather than criminal.

### Recommended changes from stakeholders

Table 4 displays two themes associated with stakeholders’ recommended policy and procedural changes.

**Table 4:** Themes of recommended changes, as discussed by focus groups of stakeholders

*Themes*	*Code*	*Example quotes from each focus group (FG)*
**Changes in implementation and enforcement protocol are needed**	**Frequency of compliance checks**	*‘I think three checks a year sounds fantastic to me for each retailer. We're lucky if they get one, and within two years, I mean, and that has been even better, like I said before, in the last two years, three years we've seen way more enforcement that we did before, probably because of the issues around vapes as well, when Juuls and all that hit the market, it became a huge issue.’ (FG 1)*
		*‘Ideally, we say twice a year, and one of these checks would include looking at signage and placement.’ (FG 2)*
	**Retailer education**	*‘...having some more understanding of individual needs of different retailers. We see that also in our area with vape retailers versus traditional tobacco retailers, but also with our varying needs, whether they're a QuikTrip or a mom-and-pop shop, or if they're rural versus our urban areas. It’s very, very different what their needs are, and we have rural areas that are begging for information on how to check for fake IDs, they're dealing with fakes in an amazing amount.’ (FG 1)*
		*‘I think tobacco shops and most people are well trained in those locations, but convenience stores is where there seems to be more turnover. And she said they went online that it was difficult to tell if people were taking them because you don't know if they're just turning them on and it's on their screen.’ (FG 4)*
	**Cooperating individual training and presentation during compliance checks**	*‘But they keep the same decoys year after year. And so [laughter] I was like, “Well, I mean, I’m from a small town in Arkansas of 5000 people. And if I was hired to do that, as soon as I walked in a gas station, everybody would know like, ‘Don’t you dare sell it to that …’ ” everybody knows who they are.’ (FG 4)*
		*‘It seems clear that without allowing the decoys to carry IDs, genuine IDs with real age, there’s too great a chance, at least in Colorado, we’ve seen this nationally I think as well, that the merchants will rely on the, “I don’t have it”, as a cue that this person shouldn’t be sold to.’ (FG 3)*
	**Pattern of compliance checks**	*Random: ‘It needs to be unpredictable so they're not expecting it. And actually, I have known of retailers that called the business a few miles down the road and said, "Oh, by the way, I just had my inspection." A lot of times they forewarn each other. Which really … you're losing your purpose.’ (FG 1)*
		*Propensity: ‘... also more frequent checks for those who have been found in non-compliance. They should get more follow-ups.’ (FG 5)*
	**Enforcement agency and community buy-in**	*‘But enforcement officers are hesitant to enforce things that would be too burdensome, even when they're appropriate.’ (FG 1)*
		*‘Depending on the hearing officers, they have a different outcome. So sometimes, they're getting fined. Sometimes they are getting closed down for a period of time. And sometimes, a lot of times, nothing happens at all. And I've heard anecdotally that that also then kind of makes the health departments not really interested, either not interested in performing their compliance checks beyond whatever the minimum is that they're required to do, or when they do have a violation, they don't bother reporting that violation because they feel like it's just going to be more work than it's worth if the Alcohol and Tobacco Commission isn't going to do anything to the retailer.’ (FG 4)*
		*‘And so as a community level, I don't see the support for the compliance checks anymore. They're saying, “Yeah, just leave those up to the FDA”, even, yeah, within our own tobacco control as a state, I'm not seeing the support from compliance checks anymore.’ (FG4)*
	**Who should enforce Tobacco 21?**	*‘We spend a lot of time thinking about who enforces. Is it law enforcement or is it local public health? And we in our community advocated for it to be local public health, that this is not something that law enforcement necessarily needs to spend their precious time and resources doing and that generally, I think that that was if you have the supportive local public health agency that that message, “We’re not putting an additional burden on law enforcement”, is very well received in the community.’ (FG 5)*
		*As a cost-saving measure: ‘I’m just … we almost always try to get the inspections done by the health department.’ (FG 5)*
**Penalties for retail violations should be effective**	**Loss of retail license**	*‘I think one of the key things besides just having a monetary penalty, which to them, they may look at as just the cost of doing business, is to have the suspension or revocation looming over them. Not being able to sell for a week or two weeks, that’s going to it them in the pocketbook much more than any fine will.’ (FG 5)*
		*‘We take away their license, threaten to take away their ability to sell, really that’s how you’re going to see change. It’s a privilege to sell this deadly, toxic product, but that’s how you’ll see change.’ (FG 2)*
	**Monetary penalties**	*‘I do think that fines have the teeth that are needed. It's just when it can be paired with multiple checks, I think a warning letter's great, then a fine. But if you're looking at one check a year, I think you have to consider fines for the business itself, for the business owner, right off the jump just because you don't have the time to come back in three weeks and check again to see if they have changed.’ (FG 1)*
		*‘If the fine is not large enough, then it a lot of the retailers can just look at it as a cost of doing business if they're making more money off underage sales than they will paying any fines.’ (FG 1)*
	**Penalty to business owner, rather than the clerk**	*‘I'd say on a perspective of the one thing that we do not agree with in our state is it is the worker, the individual that gets cited a ticket instead of having it go against the business, the company. So it's that hourly-rate employee that's getting a ticket and needing to go to court or whatever for doing that instead of holding the business accountable.’ (FG 1)*
		*‘I feel that if they have multiple failures, then that means we have to address that. We have to talk to the store owners. I do agree that it has to be the owners and the managers and not so much the employees or the checkers.’ (FG 2)*
		*‘They may get up on the pulpit there and say, Well, we're very rigid, and we'll fire anybody that sells. Well, they know they can … it's pretty much a revolving door on clerk turnover anyway. So that's just kind of empty words with them.’ (FG 5)*
	**Clerk education if a retail violation is made**	*‘What I’ve observed is that, usually, if the clerk is attending the class to resolve a citation that they received, that they are not paid, but it’s in lieu of a $300 fine, so usually winds up being a good deal for the clerk.’ (FG 2)*

*Theme 7: Changes in implementation and enforcement protocol are needed*

When asked about what changes are needed to improve T21 implementation and enforcement, stakeholders gave several suggestions pertaining to retail compliance check protocols, retailer education efforts, and public engagement with T21. Stakeholders had mixed opinions on the frequency of retailer compliance checks. However, the majority cited that two or three times a year would be sufficient. Stakeholders believed that the individuals completing the checks needed to change from year to year and carry IDs. Stakeholders were split on the pattern of compliance checks as some advocated for random checks, while others advocated for propensity-based checks or more frequent checks for retailers near youth-serving institutions, such as schools. When asked who they thought should complete retail compliance checks, several stakeholders reported that local health departments would be ideal. However, in order for this to be effective, they would need to have the authority to issue penalties.

Several stakeholders believed that current retail education efforts were not tailored appropriately and in order to be more effective, retail education must meet the needs of individual retailers based on the type of store and location. As explained by a stakeholder:

*‘... having more understanding of individual needs of different retailers. We see that also in our area with vape retailers versus traditional tobacco retailers, but also with our varying needs, whether they’re a QuikTrip or a mom-and-pop shop, or if they’re rural versus our urban areas.’* (Focus group 1)

A final change that stakeholders mentioned was the need to engage and increase enforcement agency and community buy-in for the T21 law. Stakeholders believed that efforts to engage and educate enforcement agencies and communities about the importance of the T21 law were important.

*Theme 8: Penalties for retail violations should be effective*

Throughout each focus group, stakeholders expressed that penalties for retail violations needed to be effective deterrents and provided suggestions of what they believed would be the most effective. In areas where tobacco retail licenses were issued, the majority of stakeholders believed that an effective policy would be to suspend the license in the first offence and then revoke the license in the case of repeated offences. In terms of monetary penalties, stakeholders believed that the fines needed to be large enough to act as a deterrent.

Stakeholders strongly advocated that in the case of a retail violation, any monetary fine should be charged to the owner of the business rather than the clerk that sold the tobacco product. It was also suggested that if a retail violation was made, rather than a monetary fine, the clerk should be offered the opportunity to complete a retailer education diversion program. In practice, some stakeholders suggested that a monetary fine for the business owner and the option to attend a diversion program in lieu of a monetary fine for the clerk, should be assessed and compared for effectiveness.

## DISCUSSION

The findings of this study provide an emic view of the strategies and challenges of T21 implementation, enforcement and outcomes from a diverse body of T21 stakeholders. To our knowledge, this is the first study examining stakeholder attitudes toward T21 implementation and enforcement. The majority of methods used by stakeholders were passive strategies, which may signify the need to incorporate more active education strategies, such as direct outreach to tobacco retailers, that could be supplemented with current educational materials. The findings from this study suggest further evaluation is needed of the different retailer education strategies, including how to improve current education programs, such as the inclusion of adult learning principles or tailored retailer education offered in several modalities (inperson, videos, e-modules). Most stakeholders also identified a lack of funding and the need for increased public awareness as barriers to their education and awareness goals. Furthermore, customized educational resources are imperative for different communities, in addition to widely applicable initiatives like the FDA’s ‘This Is Our Watch’ program^22^. This gap can be filled by additional support from the federal level, including funding for items such as custom signage tailored to a specific community’s needs as well as national efforts to raise public awareness of the T21 law.

Barriers to implementation identified by stakeholders also included state and local laws inconsistent with federal law and a lack of statewide retail licenses, both of which need to be addressed and revised at the policy level. Competing laws could result in confusion as retailers were required to post signage that had conflicting information, and local communities were unsure of which law was, in fact, the superseding law. As federal law supersedes state and local laws, state and local laws should have updated MLSAs to reflect the national minimum age of 21 years to eliminate any confusion caused by competing laws. Similarly, in order to effectively regulate T21 enforcement, comprehensive lists of tobacco retailers must be readily available, which most stakeholders identified as a key resource needed in order to properly implement T21 in their communities. While prior studies have been conducted to successfully determine the total number of tobacco retailers within a state that did not have a tobacco licensing law, statewide tobacco retail licensing laws would ensure each state would have a standardized list of all retailers as well as facilitate further regulation of retail violations when necessary^23^. Currently, 29 US states have instituted mandatory tobacco retailer licenses, which have been shown to reduce youth tobacco use and youth e-cigarette initiation^24-27^.

Vape and tobacco shops are areas of particular concern for T21 enforcement because e-cigarettes are not regulated similarly to other tobacco products. The online space was also mentioned as a major concern for enforcement as youth under the age of 21 years use e-cigarettes more than any other tobacco product and are most likely to purchase their e-cigarette devices either in vape shops or online. Further study and policy development responding to the challenges in regulating online retailers is important^28,29^ .

While not explicitly framed as an issue of equity by stakeholders, equity concerns were raised around penalties incurred by the clerk or retailer in businesses where a retail violation took place. From an equity perspective, while monetary fines would need to be enough to deter future retail violations, the size of the business in question may also need to be taken into consideration as a single locally owned business may not have similar resources as larger chain retailers.

### Limitations

There are limitations to note. First, participants of these focus groups represented 16 US states, which, given the variability of T21 implementation and enforcement based on location, may have influenced the results of this study. Second, 10 participants (32.3%) resided in Nebraska, which may have introduced a geographical bias in the results of this study. To minimize geographical bias, participants from differing states were encouraged to provide their unique perspectives. Third, we did not analyze results based on participant characteristics, such as their location or stakeholder category; however, this should be considered in future studies with large sample sizes.

### Implications

T21 implementation and enforcement varied based both on state and local policies as well as locally available resources. Most stakeholders predominantly employed passive strategies, lacking direct interaction with tobacco retailers or the general public. This suggests that there may be a necessity to adopt more active strategies that involve direct engagement with retailers. Policy changes to strengthen and align federal, state, and local efforts are needed to reduce barriers in T21 implementation and enforcement, and proactively identify and mitigate the exacerbation of existing health inequities.

## CONCLUSIONS

While the T21 law has been estimated to save over 230000 lives, key stakeholders have identified barriers threatening to weaken its impact across the United States. Our findings will allow additional research and policy development to strengthen and improve the impact on public health of the Tobacco 21 law. While T21 will not be a panacea for reducing youth tobacco use, reducing barriers to its implementation and enforcement could strengthen its impact.

## Data Availability

The data supporting this research are available from the authors on reasonable request.
